# Association between retinal vascular fractal dimension and hearing loss: a cross-sectional study

**DOI:** 10.1038/s41598-025-16451-1

**Published:** 2025-08-19

**Authors:** Rui Xu, Xing Qi, Yihan Li, Xiyun Bian, Xiaowei Zhang, Jing Wang, Tingli Chen, Xiangming Meng

**Affiliations:** 1 Department of Otolaryngology, Shanghai Health and Medical Center CN, No. 67 Dajishan, Wuxi, 214065 People’s Republic of China; 2 Department of Otolaryngology, Affiliated Huishan Hospital of Medical College, Yangzhou University, Wuxi Huishan District People’s Hospital, Zhanqian North Road, Luoshe Town, Huishan District, Wuxi, 214187 People’s Republic of China; 3 Department of Ophthalmology, Shanghai Health and Medical Center CN, No. 67 Dajishan, Wuxi, 214065 People’s Republic of China

**Keywords:** Hearing loss, Fractal dimension, Retina, Microcirculation, Health checkup, Eye diseases, Diagnostic markers, Biomarkers, Diseases

## Abstract

Hearing loss (HL) is a prevalent health issue, and identifying noninvasive biomarkers for early detection is crucial. This study investigates the association between retinal vascular fractal dimension (FD) and HL in a health checkup population. A retrospective cross-sectional study was conducted with 575 participants who underwent ophthalmological and audiological assessments at Shanghai Health and Medical Center CN between October 2014 and January 2024. Retinal FD was measured using nonmydriatic fundus photography and analyzed using Singapore I Vessel Assessment software. Hearing thresholds were assessed by air-conduction pure-tone audiometry, with the high Fletcher index (hFI) used to evaluate hearing acuity. Results showed that higher retinal FD values were significantly associated with better hearing acuity. Specifically, each 1-SD increase in arteriolar FD (FDa) was correlated with a 2.85 dB decrease in the hearing threshold at 1 kHz (β = −2.85, 95% CI − 4.47 to − 1.23, *p* < .001), and each 1-SD increase in venular FD (FDv) was correlated with a 2.61 dB decrease at 2 kHz (β = −2.61, 95% CI − 4.42 to − 0.80, *p* = .005). The study suggests that retinal FD may serve as a noninvasive biomarker for early detection of HL and could assist in the development of preventive strategies.

**Supplementary Information:**

The online version contains supplementary material available at 10.1038/s41598-025-16451-1.

## Introduction

Hearing loss (HL) represents a widespread public health concern that, without proper intervention or adequate communication support, may negatively influence diverse areas of an individual’s life^1^. HL can limit opportunities for verbal communication, thus impeding speech development in children and elevating the risk of dementia and cognitive decline in older adults^2–4^. A 2024 report from the World Health Organization indicates that around 430 million individuals globally experience HL, representing more than 5% of the world’s population, and they are categorized as needing rehabilitation due to disability^5^. By 2050, the number of people with HL is expected to reach 2.45 billion, reflecting a 56.1% increase from 2019, even though the age-standardized prevalence remains stable^1^. These statistics highlight the global burden of HL and the urgent need for early detection and intervention to improve quality of life and health.

The causes of HL are multifactorial, encompassing noise exposure, aging, genetic predisposition, ototoxic effects, nutritional factors, lipid metabolism issues, diabetes mellitus (DM), and various other influences^6–9^. Moreover, viral infections like cytomegalovirus, measles, varicella-zoster virus, and mumps can lead to sensorineural HL through direct damage to cochlear structures and immune-mediated responses^10^. Recently, COVID-19-related infections have also been associated with potential HL, with evidence suggesting that SARS-CoV-2 may affect auditory function by impacting middle ear structures^11–13^.

Recent studies have provided evidence that microvascular pathology could contribute to HL^14^. Cochlear microvasculature contains a substantial population of pericytes that participate in the pathophysiological processes of cochlear vasculature and affect the pathophysiology of various types of HL^15^. The stria vascularis, a key structure in the cochlea, plays a central role in sustaining the electrochemical environment necessary for hearing^16^. Microvascular irregularities in the stria vascularis are frequently associated with vascular disease, diminishing blood flow, impairing ion transport, and decreasing endocochlear potential. The disruption of cochlear homeostasis adversely affects the functionality of sensory hair cells, leading to progressive hearing loss typical of HL^16,17^.

The retinal fundus provides a noninvasive means of assessing human microvasculature in vivo^18^. Due to the anatomical and physiological similarities between retinal microvasculature and other body microvessels, it is frequently used as a surrogate marker for systemic microcirculation in population-based research^19^. Prior studies indicate that microvascular changes in the retinal fundus are linked to several systemic conditions, such as diabetes, acute decompensated heart failure, preclinical Alzheimer’s disease, and obstructive sleep apnea syndrome^18,20–22^. By analyzing retinal microvascular changes, researchers can identify high-risk populations for various chronic conditions early, facilitating preventative measures.

Previous studies have identified a correlation between retinal microvascular abnormalities and HL, indicating that common vascular pathologies may affect the blood flow of both the retina and the cochlea, potentially contributing to hearing loss in older individuals^23–25^. Nevertheless, these investigations primarily focused on individual retinal vessel parameters, including the arteriolar-to-venular diameter ratio (AVR), central retinal arteriolar equivalent (CRAE), and central retinal venular equivalent (CRVE), without offering a thorough assessment of retinal microcirculatory patterns^23–25^. In contrast, FD comprehensively evaluates vascular branching intricacy and density. A reduced FD value indicates a less dense vascular network, potentially reflecting significant microvascular changes associated with systemic illnesses^18,26^. Using FD as a comprehensive indicator can capture microvascular pattern changes not reflected by traditional metrics, potentially revealing systemic vascular changes relevant to HL.

The present study aims to investigate the association between FD and HL in a cross-sectional health checkup population. These findings may elucidate the underlying microvascular mechanisms associated with HL and highlight FD as a noninvasive screening tool, paving the way for future personalized therapies.

## Methods

### Study population

We conducted a retrospective investigation using data from Shanghai Health and Medical Center CN (Huadong Sanatorium), a well-established tertiary care hospital in China renowned for its healthcare services and comprehensive health checkup programs^18^. The electronic medical records of participants who underwent hearing tests from October 2014 to January 2024 were reviewed in this retrospective analysis. The information was meticulously organized into a predetermined Microsoft Excel spreadsheet during the data extraction procedure.

Participants were eligible for the study provided they fulfilled the following criteria: (1) adults aged 18 years or older, (2) who freely consented to participate, (3) having completed both a hearing assessment and a fundoscopic examination, and (4) capable of cooperating in obtaining satisfactory fundus pictures. Participants were excluded if they met any of the following criteria: (1) Presence of retinal diseases (e.g., diabetic retinopathy, age-related macular degeneration); (2) Diagnosis of glaucoma; (3) Presence of keratoconus; (4) Refractive errors > ± 6.00 diopters; (5) Acute or chronic middle ear disorders; (6) History of significant noise exposure.

Data were collected on demographic characteristics, including age, gender, smoking status, alcohol consumption, and medical conditions such as hypertension, coronary heart disease (CHD), DM, and hyperlipidemia (HYP).

### Ethical statement

The study adhered to the principles of the Declaration of Helsinki and received approval from the Medical Ethics Committee of Shanghai Health and Medical Center CN (Grant number: 2024019) and adhered to the principles outlined in the Helsinki Declaration and other relevant ethical standards. All participants provided written informed consent for the use of their data. Personal information was anonymized before analysis, and strict confidentiality was maintained throughout the statistical process. The data were used solely for scientific research purposes.

### Measures

#### Retinal vessel caliber

All participants underwent nonmydriatic fundus photography, with images captured using a no-dilatation fundus camera (NW400; Topcon Corporation, Tokyo, Japan) centered on the optic disc and covering a 45° field of view. These photographs were then quantitatively analyzed by two trained ophthalmologists using the Singapore I Vessel Assessment (SIVA) software (version 4.0; School of Computing, National University of Singapore, Singapore), specifically for the right eye. In cases where the right eye’s fundus image was deemed unsatisfactory, the left eye’s image was analyzed instead. Given the inconsistencies in the software’s automatic measurements and occasional suboptimal photograph quality, manual corrections were necessary for most images. All analyses strictly followed the software’s manual instructions to ensure accuracy and reproducibility^27^.

In FD analysis, higher FD values indicate more complex retinal vascular branching patterns. Supplemental Fig. 1 illustrates that zone B covers the area from 0.5 to 1 papillary diameter (PD) from the optic disc edge, while zone C spans 1 to 2 PD. The software quantified key morphological features of retinal vessels in these zones, including arteriole FD (FDa), venule FD (FDv), and total FD.

To facilitate understanding of the FD concept, we included representative fundus photographs from participants with high, moderate, and low FD values in Supplemental Fig. 2. These images illustrate differences in retinal vascular complexity, with higher FD values corresponding to more complex and denser vascular networks.

#### Audiometry

Air conduction pure-tone audiometry was performed by trained examiners using a computer-based audiometer (Model 1066, Otometrics A/S, Denmark) with headphones, following a standardized modified Hughson-Westlake audiometric technique^28^. Testing commenced at 20 dB HL for the initial frequency (1 kHz); subsequent frequencies were tested at 10 dB HL if thresholds were within the normal range. This approach allowed for a thorough assessment of hearing thresholds across various frequencies. HL was defined as a pure-tone average (PTA) threshold greater than 20 dB hearing level in the better-hearing ear, measured at 0.5, 1, 2, and 4 kHz, in accordance with the World Health Organization (WHO) criteria^29^.

#### Data management

The high Fletcher index (hFI), the average hearing threshold at 1, 2, and 4 kHz, was calculated for each participant because these frequencies correspond closely to the critical range of speech sounds^30^. This index provides a dependable measure of hearing ability related to speech comprehension.

### Statistical analysis

Means (standard deviations [SDs]) for continuous variables and frequencies (percentages) for categorical variables were used to describe baseline characteristics. Restricted cubic spline plots created with the R software’s rms package were used to investigate nonlinear relationships between FD and hearing thresholds. The median FD served as the reference, and models were adjusted for factors such as age, sex, smoking status, alcohol consumption, hypertension, CHD, DM, and HYP.

Multivariable linear regression models were employed to investigate the relationship between FD and hearing thresholds. β coefficients and 95% CIs were derived for each model. Three models were specified: Model 1 was unadjusted (crude); Model 2 included adjustments for age and sex; and Model 3 included additional adjustments for smoking status, alcohol consumption, hypertension, CHD, DM, and HYP.

Analyses were conducted using R (version 4.3.3; R Foundation for Statistical Computing, Vienna, Austria), with statistical significance defined as a two-tailed p-value of less than 0.05.

## Results

### Participants characteristics

From 2021 to 2024, 691 participants underwent ophthalmologic and audiologic assessments. After applying the inclusion and exclusion criteria, 116 were excluded due to ocular or auditory conditions. The final analysis included 575 participants. The STROBE flowchart illustrates the recruitment process (Supplemental Fig. 3).

Their baseline characteristics are summarized in Table 1. The mean age was 58.28 years (SD: 9.93); 66.09% (*n* = 380) were male, and 33.91% (*n* = 195) were female. The mean values for FDa, FDv, and FD were 1.12 (SD: 0.05), 1.11 (SD: 0.04), and 1.26 (SD: 0.05), respectively. Additional demographic and clinical data, including smoking status, alcohol consumption, hypertension, CHD, DM, and HYP, are presented in Table 1.

**Table 1:** Demographic and clinical characteristics of study participants.

Characteristics	Total (*n* = 575)
FD (mean ± SD)	1.26 ± 0.05
FDa (mean ± SD)	1.12 ± 0.05
FDv (mean ± SD)	1.11 ± 0.04
Age, year (mean ± SD)	58.28 ± 9.93
Sex	
Male	380 (66.09%)
Female	195 (33.91%)
Smoking	
No	421 (73.22%)
Yes	154 (26.78%)
Drinking	
No	421 (73.22%)
Yes	154 (26.78%)
HP	
No	384 (66.78%)
Yes	191 (33.22%)
CHD	
No	515 (89.57%)
Yes	60 (10.43%)
DM	
No	490 (85.22%)
Yes	85 (14.78%)
HYP	
No	475 (82.61%)
Yes	100 (17.39%)

*FD* total fractal dimension, *FDa* arteriole fractal dimension, *FDv* venule fractal dimension, *HP* hypertension, *CHD* coronary heart disease, *DM* diabetes mellitus, *HYP* hyperlipidemia.

### Associations between FD and hearing acuity

**Fig. 1:**
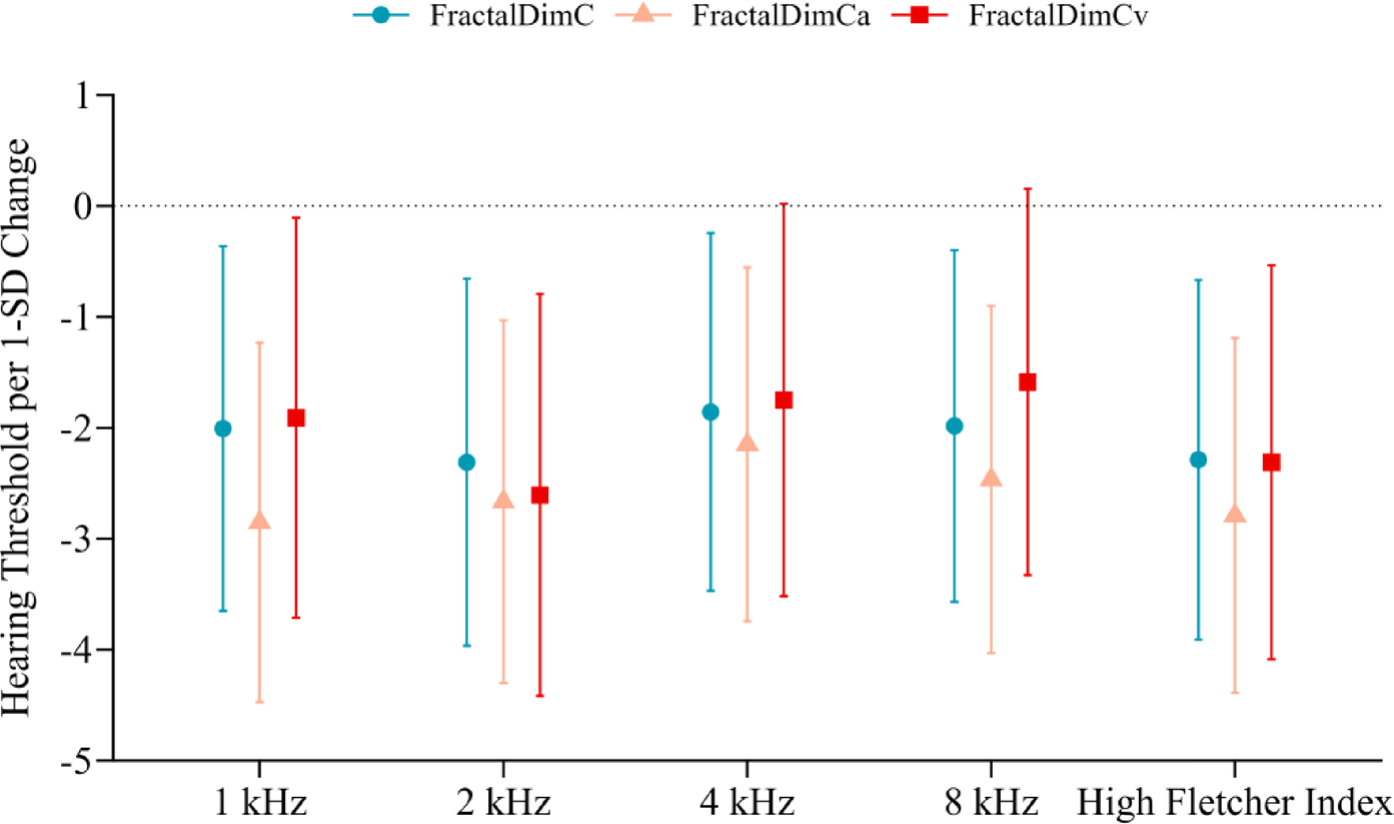
The figure depicts the relationships between changes in FD metrics (FractalDimC, FractalDimCa, and FractalDimCv) and hearing thresholds at 1 kHz, 2 kHz, 4 kHz, 8 kHz, and the hFI. The y-axis shows the change in hearing thresholds per 1-SD increase in FD, where a negative change denotes improved hearing acuity. Error bars indicate 95% confidence intervals. FractalDimC, FractalDimCa, and FractalDimCv represent the complexity of the retinal vascular network in various regions, illustrating that higher FD is linked to lower hearing thresholds across the frequency range.

The linear regression analysis results examining the associations between FD and hearing thresholds are shown in Fig. 1. In Model 3, which was fully adjusted, higher FDa and FDv values were inversely related to hearing thresholds, suggesting better hearing acuity with greater retinal vascular complexity. Specifically, a 1-SD increase in FDa corresponded to a 2.85 dB HL reduction in the hearing threshold at 1 kHz (β = -2.85; 95% CI: -4.47 to -1.23; *p* < .05) (Table 2), while a 1-SD increase in FDv corresponded to a 2.61 dB HL reduction at 2 kHz (β = -2.61; 95% CI: -4.42 to -0.80; *p* < .05) (Table 3). Likewise, total FD was negatively correlated with hearing thresholds, with a 1-SD increase leading to a 2.31 dB HL reduction at 2 kHz (β = -2.31; 95% CI: -3.97 to -0.66; *p* < .05) (Supplemental Table 1). Additionally, each 1-SD increase in FDa, FDv, and FD was linked to a decrease in hFI values, with β coefficients (95% CI) of -2.79 (-4.39 to -1.19), -2.31 (-4.09 to -0.53), and − 2.29 (-3.91 to -0.67), respectively (all *p* < .05).

**Table 2:** Associations between FDa metrics and hearing thresholds across different frequencies.

		Model1		Model 2		Model 3	
Outcome	N	β (95%CI)	P value	β (95%CI)2	P value	β (95%CI)	P value
1 kHz	575	-3.99 (-5.61, -2.37)	< 0.001	-3.56 (-5.16, -1.96)	< 0.001	-2.85 (-4.47, -1.23)	< 0.001
2 kHz	575	-3.57 (-5.19, -1.94)	< 0.001	-3.14 (-4.75, -1.53)	< 0.001	-2.67 (-4.30, -1.03)	0.001
4 kHz	575	-3.01 (-4.64, -1.38)	< 0.001	-2.43 (-4.00, -0.86)	0.002	-2.15 (-3.74, -0.55)	0.008
8 kHz	575	-3.55 (-5.18, -1.92)	< 0.001	-3.00 (-4.57, -1.44)	< 0.001	-2.47 (-4.03, -0.90)	0.002
High fletcher index	575	-3.85 (-5.47, -2.23)	< 0.001	-3.29 (-4.87, -1.71)	< 0.001	-2.79 (-4.39, -1.19)	< 0.001

Model 1 was unadjusted, without controlling for any confounding factors; Model 2 adjusted for age and gender; and Model 3 was fully adjusted for age, gender, smoking status, alcohol consumption, hypertension, CHD, DM, and HYP. The regression coefficient (β) represents the change in hearing threshold (dB nHL) per 1-SD change in FDv.

**Table 3:** Associations between Fd metrics and hearing thresholds across different frequencies.

		Model1		Model 2		Model 3	
Outcome	N	β(95%CI)	P value	β(95%CI)2	P value	β(95%CI)	P value
1 kHz	575	-3.11 (-4.95, -1.28)	< 0.001	-2.59 (-4.40, -0.78)	0.005	-1.91 (-3.71, -0.11)	0.038
2 kHz	575	-3.61 (-5.44, -1.78)	< 0.001	-3.13 (-4.94, -1.32)	< 0.001	-2.61 (-4.42, -0.80)	0.005
4 kHz	575	-2.67 (-4.50, -0.83)	0.004	-2.18 (-3.95, -0.41)	0.016	-1.75 (-3.52, 0.02)	0.052
8 kHz	575	-2.66 (-4.50, -0.83)	0.004	-2.22 (-3.99, -0.46)	0.014	-1.59 (-3.33, 0.15)	0.074
High fletcher index	575	-3.45 (-5.28, -1.62)	< 0.001	-2.89 (-4.68, -1.11)	0.001	-2.31 (-4.09, -0.53)	0.011

Model 1 was unadjusted, without controlling for any confounding factors; Model 2 adjusted for age and gender; and Model 3 was fully adjusted for age, gender, smoking status, alcohol consumption, hypertension, CHD, DM, and HYP. The regression coefficient (β) represents the change in hearing threshold (dB HL) per 1-SD change in FDv.

To complement the regression findings, Supplementary Fig. 4 presents scatter plots that show the relationship between individual FD values and hFI, which illustrate the negative linear associations.

Age was a significant factor influencing both FD and HL. Nevertheless, partial correlation analyses revealed that, after adjusting for age, arteriolar FD (FDa, *p* = .004), venular FD (FDv, *p* = .023), and total FD (*p* = .014) remained significantly associated with HL. These findings indicate that the observed associations between FD and HL are not solely attributable to age-related changes (Supplemental Table 2).

### Restricted cubic spline regression analysis

**Fig. 2:**
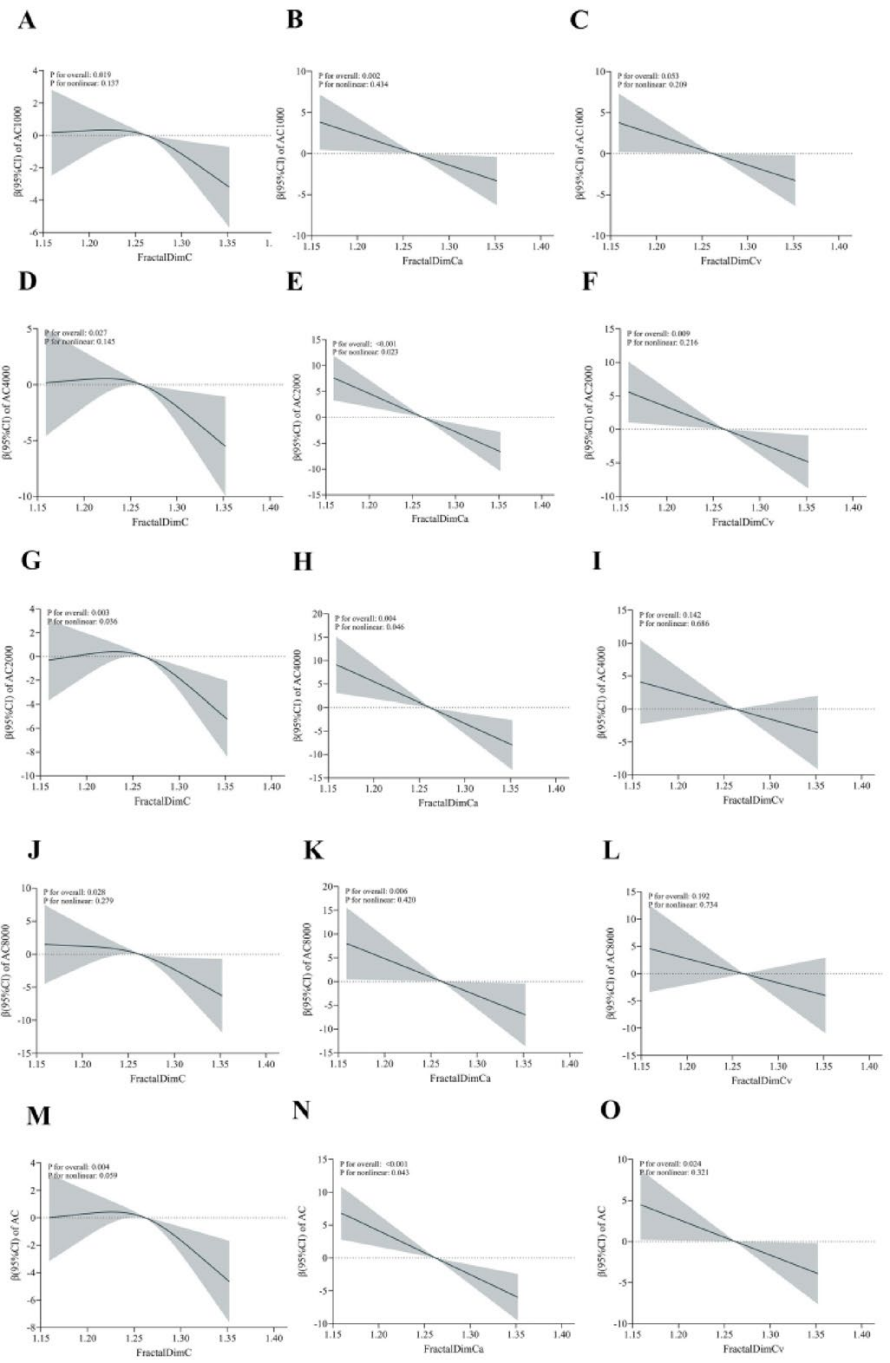
This t illustrates the results of restricted cubic spline regression analysis assessing the associations between fractal dimension metrics (FractalDimC, FractalDimCa, and FractalDimCv) and hearing loss at different frequencies: 1 kHz, 2 kHz, 4 kHz, 8 kHz, and across the hFI. Panels (**A**–**C**) show the associations at 1 kHz, where FractalDimCa and FractalDimCv demonstrate significant inverse relationships with hearing thresholds. Panels (**D**–**F**) present the results at 2 kHz, highlighting a pronounced negative association for FractalDimCv. Panels (**G**–**I**) depict the analysis at 4 kHz, with notable inverse trends for FractalDimCa. Panels (**J**–**L**) cover 8 kHz, showing a consistent, though less pronounced, relationship for FractalDimCa and FractalDimC. Finally, Panels (**M**–**O**) summarize the hFI analysis, with all FD metrics indicating clear inverse associations. The solid lines represent central risk estimates, and the shaded areas denote 95% confidence intervals, adjusted for age, gender, smoking status, alcohol consumption, HP, CHD, DM, and HYP.

Figure 2 depicts the correlation between FD and hearing thresholds as modeled by restricted cubic spline regression. The results indicate that the β coefficients for hearing thresholds continually decline as FD increases, signifying a reduced risk of HL linked to higher FD values.

## Discussion

### Principal findings

This cross-sectional study found a significant association between FD and HL in a health checkup population. Our results indicate that higher FDa, FDv, and total FD values were associated with better hearing acuity, as reflected by lower hearing thresholds. Specifically, each 1-SD increase in FDa and FDv was associated with a 2.85 dB HL reduction at 1 kHz and a 2.61 dB HL reduction at 2 kHz, respectively. These associations remained consistent across frequencies and with the hFI. Restricted cubic spline regression confirmed that the risk of hearing loss decreased as FD values increased. These findings suggest that retinal microvascular abnormalities could serve as systemic markers involved in the pathophysiology of HL.

### Comparison with previous studies

Our findings support and expand previous research on the relationship between retinal microvascular abnormalities and HL. The Blue Mountains Hearing Study, the first international study to investigate this association, found a significant link between retinal microvascular abnormalities and HL, highlighting the importance of systemic microcirculation in hearing function^23^. Similarly, Kim et al., in the ARIC-NCS cohort, confirmed that retinal microvascular signs, such as retinopathy, were associated with hearing impairment, particularly among non-DM individuals^24^. Sardone et al. also identified an inverse relationship between retinal vascular density and central auditory processing disorders in older adults^25^. In contrast, our study uniquely used FD to comprehensively measure vascular complexity, providing a broader evaluation of retinal microvascular health and its relationship with HL across various frequencies. This approach suggests that FD may be a more sensitive marker for detecting systemic vascular changes related to HL.

### Biological or clinical mechanisms

The association between FD and HL observed in this study may be due to shared microvascular pathologies affecting retinal and cochlear circulation. Since retinal and cochlear tissues are highly vascularized and sensitive to ischemic damage, they are vulnerable to systemic microvascular dysfunction^31,32^. Reduced retinal vascular density may indicate underlying microvascular insufficiencies affecting cochlear blood flow, leading to sensory hair cell degeneration and subsequent HL^23,24^. An example supporting this association is Susac syndrome, a rare condition involving occlusive microvascular disease of the brain, retina, and inner ear, which demonstrates how microvascular pathology can impact both retinal and auditory structures^23,33^.

Another possible cause is endothelial dysfunction in microcirculatory networks, as endothelial cells play an important role in vascular tone modulation and blood flow dynamics^34^. Malfunctions in these cells can undermine the blood-labyrinth barrier in the cochlea, which is crucial for preserving the electrochemical equilibrium necessary for auditory function^35^. The disruption of this barrier could hinder the ion transport mechanisms regulated by the stria vascularis, resulting in auditory dysfunction^35^.

Furthermore, the FD of retinal vessels provides an integrated measure of vascular complexity and efficiency by quantifying the scale-invariant branching patterns of the retinal vascular network, encompassing characteristics such as bifurcation frequency, branch angles, and segment lengths^26,36^. A lower FD indicates a simplified vascular architecture with reduced branching density^26^potentially compromising tissue perfusion in the retina and cochlea. Notably, lower FD and density are associated with increased risks of mortality, cardiovascular diseases, DM, sleep apnea, and anemia^26,37^. Therefore, reduced retinal FD may serve as a marker of generalized vascular aging or early microvascular disease, linking systemic vascular health to sensory impairments like HL^23,24^.

In addition to the hypotheses previously discussed, we propose two additional testable mechanisms that may explain the observed association between reduced retinal vascular FD and HL.

First, a deficit in neurovascular coupling: recent findings from brain studies indicate that pericytes along capillary walls actively regulate vessel tone and local blood flow in response to neuronal activity^38^. Loss or dysfunction of these pericytes can impair neurovascular coupling and reduce oxygen delivery. A similar mechanism may exist in the cochlea, where pericytes are densely distributed in the stria vascularis and spiral ligament, contributing to the regulation of cochlear blood flow^15^. Disruption of pericyte-mediated regulation in the cochlear microvasculature may result in transient ischemia, potentially accelerating HL.

Second, a deficit in glymphatic-like clearance: the cochlea has been reported to contain a glymphatic-like system that removes metabolic waste (e.g., amyloid-β) via paravascular fluid transport, similar to the brain’s glymphatic pathway^39^. As indicated by lower retinal FD, a reduction in systemic microvascular complexity may compromise this clearance mechanism, leading to toxin accumulation and damage to auditory structures. These mechanisms provide biologically plausible links between retinal and cochlear microvascular health and warrant further investigation in longitudinal studies.

### Strengths and limitations

To our knowledge, this is the first study using FD as a metric to examine the relationship between retinal vascular characteristics and hearing loss across low and high frequencies. FD provides a broader evaluation of retinal microvascular health, capturing the overall complexity of vascular branching patterns, which traditional metrics such as CRAE, CRVE, and AVR, primarily focused on vessel diameter, may fail to capture adequately^36^. Previous studies have shown that lower FD and density are significantly associated with increased risks of mortality, hypertension, stroke, congestive heart failure, renal failure, DM, sleep apnea, and anemia^26,38^. By analyzing FD, we captured broader patterns of vascular complexity that may play a role in the pathophysiology of HL. These patterns might be overlooked when examining only specific vessel calibers. Additionally, the relatively large sample size from a routine health checkup population improves the generalizability of our findings, supporting their relevance in non-clinical settings. Another strength is the rigorous statistical adjustment for potential confounders, including age, sex, smoking, alcohol consumption, and chronic conditions such as hypertension, coronary heart disease, DM, and HYP, which enhances the robustness of our results.

This study has several limitations. Firstly, the cross-sectional design restricts our ability to establish causal relationships between retinal vascular changes and ARHL, leaving it uncertain whether these microvascular alterations precede or follow hearing loss. Longitudinal studies are needed to clarify this temporal association. Secondly, residual confounding variables such as nutrition, physical activity, and medication usage may persist despite adjustments for major confounders. Thirdly, the absence of bone conduction audiometry limits our capacity to differentiate between sensorineural and conductive HL. Lastly, although the observed effect sizes were small, even modest associations may hold clinical relevance at the population level.

### Future directions

This study highlights the association between FD and HL, although further investigation is needed. Longitudinal studies are critical to establish causation and determine whether retinal microvascular changes occur before HL. Second, future studies should incorporate bone conduction audiometry to distinguish between sensorineural and conductive HL. Third, exploring the management of systemic conditions such as hypertension and diabetes and their effects on HL is valuable, as is investigating lifestyle factors such as diet and physical activity^40,41^. Finally, advanced imaging techniques such as optical coherence tomography angiography could offer more precise retinal vascular assessments and a more detailed understanding of microvascular changes associated with HL^42^.

## Conclusions

In conclusion, this study reveals a significant association between FD and HL, where higher values of FDa, FDv, and total FD correlate with better hearing acuity. These findings imply that retinal microvascular abnormalities may act as systemic indicators of the underlying pathophysiology of hearing loss. Retinal microvascular assessments may hold promise for the early detection and intervention of hearing impairment, supporting the development of more effective prevention and treatment strategies.

## Supplementary Information

Below is the link to the electronic supplementary material.

Supplementary Material 1

Supplementary Material 2

Supplementary Material 3

Supplementary Material 4

Supplementary Material 5

## Data Availability

The datasets used and/or analyzed during the current study are available from the corresponding author or co-author on reasonable request.

## Abbreviations

HL: Hearing loss
CRAE: Central retinal arteriolar equivalent
CRVE: Central retinal venular equivalent
AVR: Arteriolar-to-venular diameter ratio
FD: Fractal dimension
CHD: Coronary heart disease
DM: Diabetes mellitus
HYP: Hyperlipidemia
SIVA: Singapore I vessel assessment
PD: Papillary diameter
FDa: Arteriole fractal dimension
FDv: Venule fractal dimension
hFI: High fletcher index
CIs: Confidence intervals
SD: Standard deviation
