# Supplementary material for: Association between retinal vascular fractal dimension and hearing loss: a cross-sectional study
Source: Sci Rep. 2025 Aug 19;15:30425. doi: 10.1038/s41598-025-16451-1 (PMC12365288; doi:10.1038/s41598-025-16451-1)
Supplement: Supplementary file 1 — Supplementary Material 1 [file 41598_2025_16451_MOESM1_ESM.docx]

**Supplemental Figure 1**


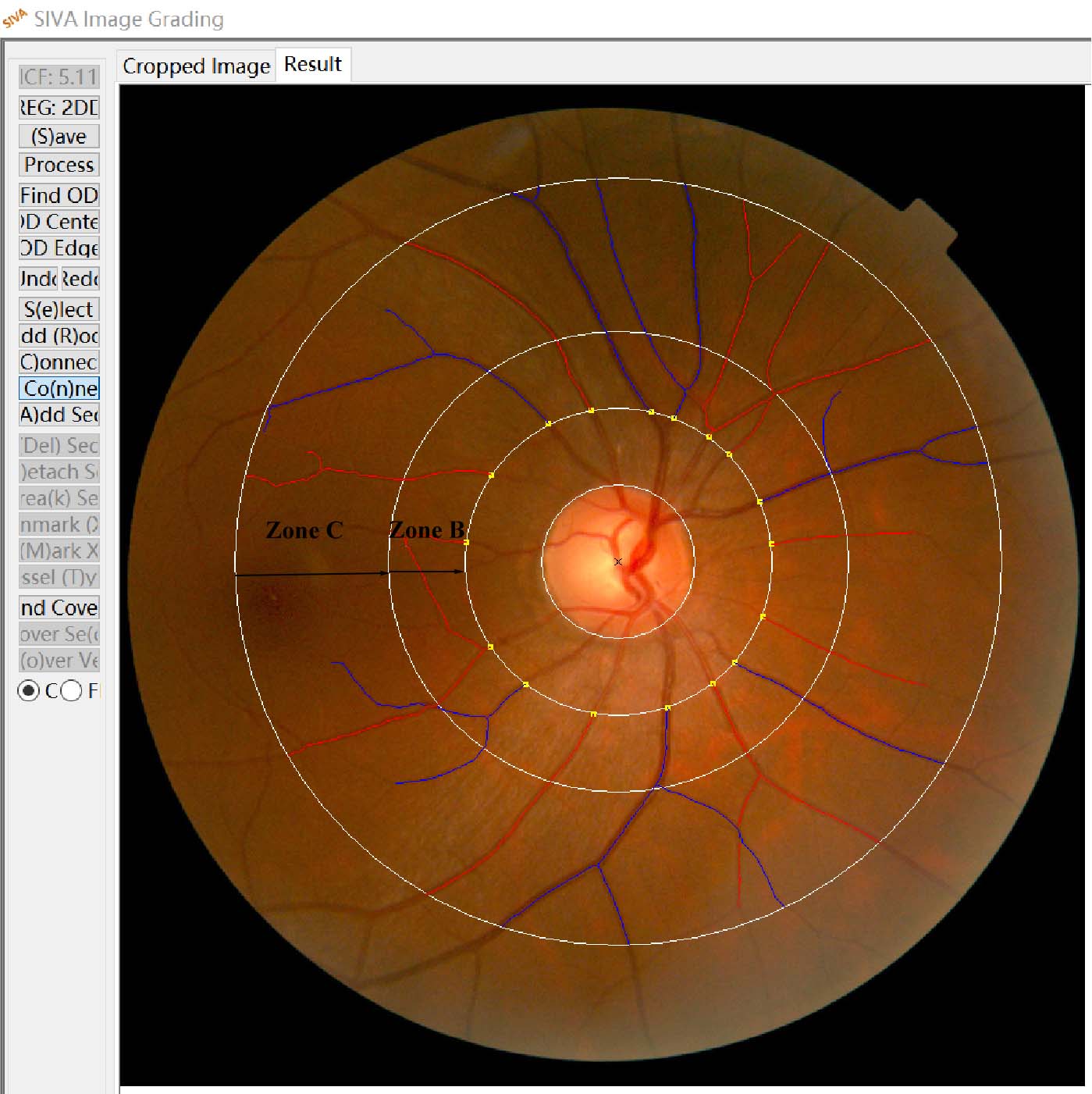


Supplemental Figure 1. The figure displays the operating interface of the Singapore I Vessel Assessment (SIVA) software (version 4.0, School of Computing, National University of Singapore) used to measure retinal vascular FD. Zone B covers the area from 0.5 to 1 PD from the optic disc edge, while zone C extends from 1 to 2 PD from the optic disc edge.
