# Supplementary material for: Association between retinal vascular fractal dimension and hearing loss: a cross-sectional study
Source: Sci Rep. 2025 Aug 19;15:30425. doi: 10.1038/s41598-025-16451-1 (PMC12365288; doi:10.1038/s41598-025-16451-1)
Supplement: Supplementary file 2 — Supplementary Material 2 [file 41598_2025_16451_MOESM2_ESM.docx]

**Supplemental Figure 2**


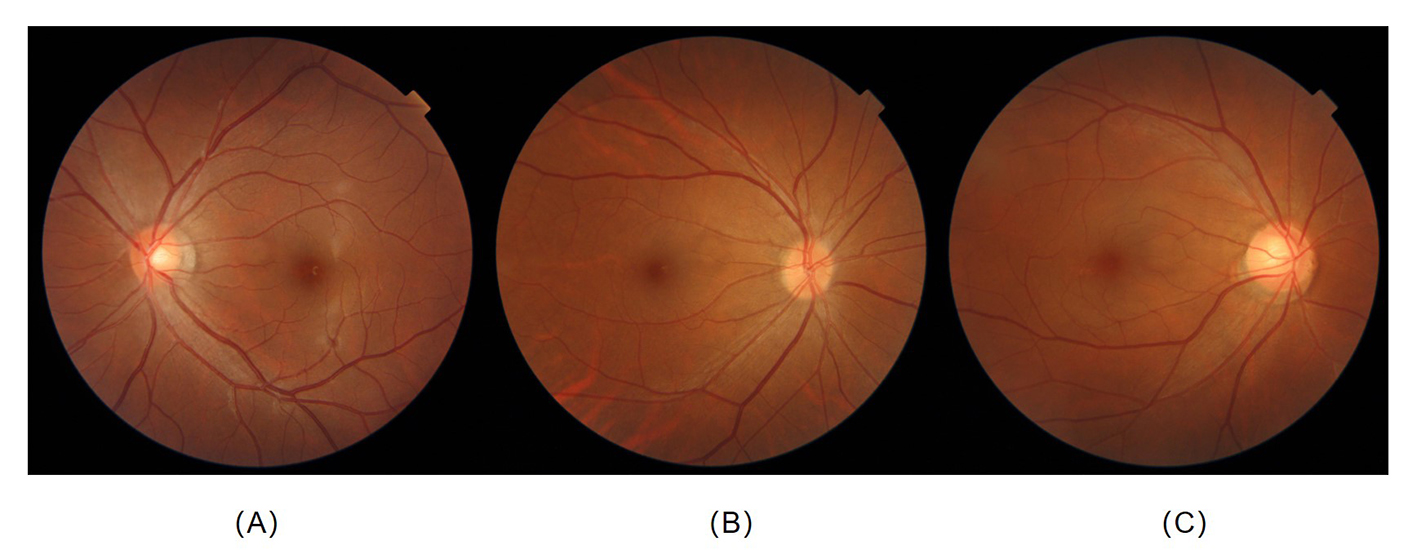


**Supplementary Figure 2. Representative Fundus Photographs Illustrating Varying Levels of Retinal Vascular Fractal Dimension (FD)**

(A) **High FD:** A dense and highly branched retinal vascular network, corresponding to a high FD value.
(B) **Moderate FD:** A retinal vascular pattern of intermediate complexity, corresponding to a moderate FD value.
(C) **Low FD:** A sparse and less branched vascular network, corresponding to a low FD value.
