# Supplementary material for: Association between retinal vascular fractal dimension and hearing loss: a cross-sectional study
Source: Sci Rep. 2025 Aug 19;15:30425. doi: 10.1038/s41598-025-16451-1 (PMC12365288; doi:10.1038/s41598-025-16451-1)
Supplement: Supplementary file 3 — Supplementary Material 3 [file 41598_2025_16451_MOESM3_ESM.docx]

**Supplemental Figure 3**


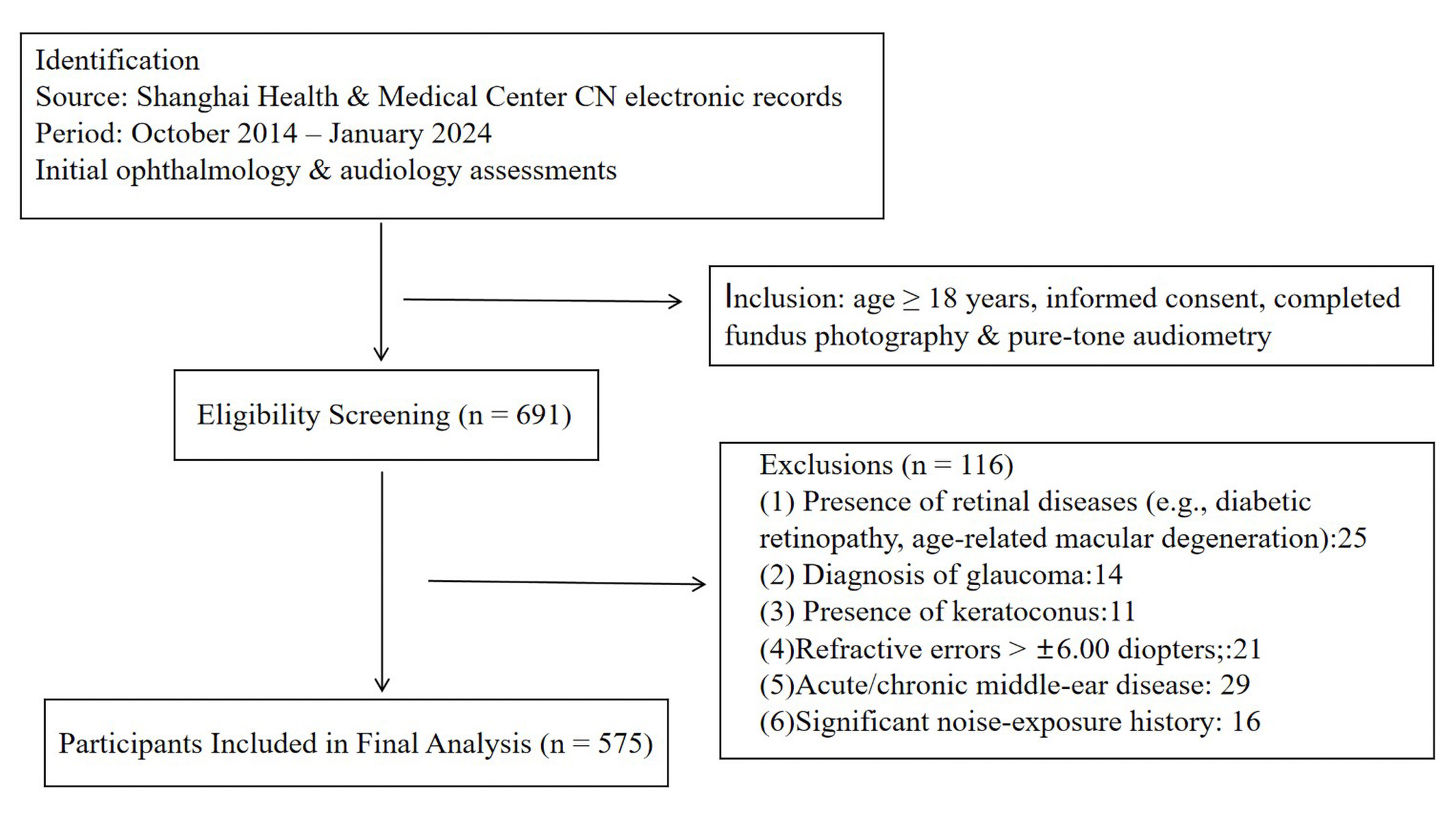


**Supplemental Figure 3. STROBE flowchart illustrating participant selection.**A total of 691 individuals who underwent ophthalmologic and audiologic assessments between October 2014 and January 2024 were screened for eligibility. Inclusion criteria were age ≥ 18 years, informed consent, and completion of both fundus photography and pure-tone audiometry. A total of 116 individuals were excluded due to ocular or auditory conditions, including retinal diseases (n = 25), glaucoma (n = 14), keratoconus (n = 11), high refractive errors (±6.00 diopters; n = 21), middle ear disease (n = 29), and a history of significant noise exposure (n = 16). The final analysis included 575 eligible participants.
