# Supplementary material for: Association between retinal vascular fractal dimension and hearing loss: a cross-sectional study
Source: Sci Rep. 2025 Aug 19;15:30425. doi: 10.1038/s41598-025-16451-1 (PMC12365288; doi:10.1038/s41598-025-16451-1)
Supplement: Supplementary file 4 — Supplementary Material 4 [file 41598_2025_16451_MOESM4_ESM.docx]

**Supplemental Table 1：Associations Between FD Metrics and Hearing Thresholds Across Different Frequencies**

|  |  | Model1 | | Model 2 | | Model 3 | |
| --- | --- | --- | --- | --- | --- | --- | --- |
| **Outcome** | **N** | **β(95%CI)** | **P value** | **β(95%CI)2** | **P value** | **β(95%CI)** | **P value** |
| 1 kHz | 575 | -3.21 (-4.87, -1.55) | <0.001 | -2.75 (-4.39, -1.11) | 0.001 | -2.01 (-3.65, -0.36) | 0.017 |
| 2 kHz | 575 | -3.27 (-4.93, -1.62) | <0.001 | -2.84 (-4.48, -1.20) | <0.001 | -2.31 (-3.97, -0.66) | 0.006 |
| 4 kHz | 575 | -2.74 (-4.41, -1.08) | 0.001 | -2.22 (-3.82, -0.62) | 0.006 | -1.86 (-3.47, -0.24) | 0.024 |
| 8 kHz | 575 | -3.08 (-4.73, -1.42) | <0.001 | -2.59 (-4.19, -1.00) | 0.001 | -1.98 (-3.57, -0.40) | 0.014 |
| High Fletcher Index | 575 | -3.40 (-5.05, -1.74) | <0.001 | -2.86 (-4.47, -1.25) | <0.001 | -2.29 (-3.91, -0.67) | 0.006 |

Notes: Model 1 was unadjusted, without controlling for any confounding factors; Model 2 adjusted for age and gender; and Model 3 was fully adjusted for age, gender, smoking status, alcohol consumption, hypertension, CHD, DM, and hyperlipidemia. The regression coefficient (β) represents the change in hearing threshold (dB HL) per 1-SD change in FD.
