# Supplementary material for: Association between retinal vascular fractal dimension and hearing loss: a cross-sectional study
Source: Sci Rep. 2025 Aug 19;15:30425. doi: 10.1038/s41598-025-16451-1 (PMC12365288; doi:10.1038/s41598-025-16451-1)
Supplement: Supplementary file 5 — Supplementary Material 5 [file 41598_2025_16451_MOESM5_ESM.docx]

**Supplemental Table 2: Correlations and Age-adjusted Partial Correlations among Fractal Dimension (FD) and Hearing loss**

| Variable | OR (95%CI) | Age-adjusted partial correlation p |
| --- | --- | --- |
| FDa | 0.60 (0.42, 0.85) | 0.004 |
| FDv | 0.64 (0.43, 0.94) | 0.023 |
| FD | 0.64 (0.45, 0.91) | 0.014 |

Notes: Odds ratios (ORs) with 95% confidence intervals (CIs) are presented for the associations between each FD metric and hearing loss. Age-adjusted partial correlation p-values are also provided. Significant inverse associations were observed for arteriolar FD (FDₐ), venular FD (FDᵥ), and total FD, indicating that lower retinal vascular complexity is associated with a higher risk of hearing loss, independent of age.
